# Biological Activity and Antidiabetic Potential of C-Terminal Octapeptide Fragments of the Gut-Derived Hormone Xenin

**DOI:** 10.1371/journal.pone.0152818

**Published:** 2016-03-31

**Authors:** Christine M. Martin, Vadivel Parthsarathy, Annie Hasib, Ming T. Ng, Stephen McClean, Peter R. Flatt, Victor A. Gault, Nigel Irwin

**Affiliations:** SAAD Centre for Pharmacy and Diabetes, Biomedical Sciences Research Institute, University of Ulster, Coleraine, Northern Ireland, United Kingdom; University of Lancaster, UNITED KINGDOM

## Abstract

Xenin is a peptide that is co-secreted with the incretin hormone, glucose-dependent insulinotropic polypeptide (GIP), from intestinal K-cells in response to feeding. Studies demonstrate that xenin has appetite suppressive effects and modulates glucose-induced insulin secretion. The present study was undertaken to determine the bioactivity and antidiabetic properties of two C-terminal fragment xenin peptides, namely xenin 18–25 and xenin 18–25 Gln. In BRIN-BD11 cells, both xenin fragment peptides concentration-dependently stimulated insulin secretion, with similar efficacy as the parent peptide. Neither fragment peptide had any effect on acute feeding behaviour at elevated doses of 500 nmol/kg bw. When administered together with glucose to normal mice at 25 nmol/kg bw, the overall insulin secretory effect was significantly enhanced in both xenin 18–25 and xenin 18–25 Gln treated mice, with better moderation of blood glucose levels. Twice daily administration of xenin 18–25 or xenin 18–25 Gln for 21 days in high fat fed mice did not affect energy intake, body weight, circulating blood glucose or body fat stores. However, circulating plasma insulin concentrations had a tendency to be elevated, particularly in xenin 18–25 Gln mice. Both treatment regimens significantly improved insulin sensitivity by the end of the treatment period. In addition, sustained treatment with xenin 18–25 Gln significantly reduced the overall glycaemic excursion and augmented the insulinotropic response to an exogenous glucose challenge on day 21. In harmony with this, GIP-mediated glucose-lowering and insulin-releasing effects were substantially improved by twice daily xenin 18–25 Gln treatment. Overall, these data provide evidence that C-terminal octapeptide fragments of xenin, such as xenin 18–25 Gln, have potential therapeutic utility for type 2 diabetes.

## Introduction

Xenin is a 25 amino acid gastrointestinal hormone, secreted from enteroendocrine K-cells in response to feeding, that performs a spectrum of biological activities [1]. As such, xenin is now known to not only effect gastrointestinal transit rate and feeding behaviour [2–5], but also acts as an independent insulinotropic agent [6,7] and reduces postprandial glucose levels in animals and humans with and without type 2 diabetes [8–10]. Interestingly, xenin may also act as a potentiator of the insulin secretory actions of the incretin hormone, glucose-dependent insulinotropic polypeptide (GIP), which is co-secreted with xenin from a subset of intestinal K-cells [7,8,10,11]. The overall physiological importance of the biological activity of xenin is highlighted by the fact that its amino acid sequence is highly conserved through evolution [12]. These various attributes suggest that xenin-based compounds could have potential application for the treatment of type 2 diabetes [8].

However, the possible therapeutic effectiveness of native xenin appears to be significantly restricted due to is efficient degradation by plasma enzymes [7,10]. In this regard, the degradation products and enzymatic cleavage sites of xenin have already been determined through use of ESI-MS/MS sequencing [10]. Notably, the C-terminal octapeptide fragment of xenin, xenin 18–25, has been identified in the circulation [1], and shown to possess insulinotropic effects in the perfused rat pancreas [13]. In agreement, our laboratory has demonstrated significant *in vitro* and *in vivo* glucose-lowering and insulin-releasing actions of this naturally occurring C-terminal xenin fragment peptide [6]. In addition, xenin 18–25 was also revealed to impart potential synergistic effects on GIP-induced insulin release [6]. Thus, it appears that the C-terminal octapeptide amino acid sequence of xenin retains bioactivity essentially similar to its parent peptide.

Interestingly, amino acid substitution of the Lys and Arg residues within native xenin with Gln, regions known to be linked to the enzymatic cleavage sites of the native peptide [6], to produce xenin-25 Gln, was recently shown to generate a remarkably potent xenin molecule [14]. As such, xenin-25 Gln exhibited a spectrum of beneficial metabolic effects in high-fat-fed and obese diabetic (*ob/ob*) mice [14]. In light of this, and increasing attention on the use of truncated and easier to synthesise fragment peptides as alternatives to the full length molecules [15], xenin 18–25 Gln could possess significant therapeutic potential for type 2 diabetes. Moreover, small molecular weight peptides could help facilitate non-injectable drug administration through appropriate formulation for oral or intransal delivery [16,17]. Therefore, in the current study we initially assessed *in vitro* insulinotropic and *in vivo* glucose-lowering, insulin releasing and satiety actions of xenin 18–25 and xenin 18–25 Gln. We then examined the beneficial effects of twice daily injection of each fragment peptide in high-fat fed mice. The results reveal that xenin 18–25 Gln is a C-terminal xenin fragment molecule that requires further consideration as a treatment option for type 2 diabetes.

## Methods

### Peptide synthesis

Native xenin, xenin 18–25 and xenin 18–25 Gln were purchased from GL Biochem Ltd (Shanghai, China, greater than 95% purity). Peptides were characterised in-house using HPLC and MALDI-TOF mass spectrometry, as described previously [10]. The experimental mass for all peptides corresponded closely to their theoretical values, confirming structural identity (data not shown). Table 1 depicts amino acid sequences of the three peptides.

**Table 1:** Amino acid sequence of xenin, xenin 18–25 and xenin 18–25 Gln.

Peptide Name	Amino acid sequence
Xenin	H-MET-LEU-THR-LYS-PHE-GLU-THR-LYS-SER-ALA-ARG-VAL-LYS-GLY-LEU-SER-PHE**-HIS-PRO-LYS-ARG-PRO-TRP-ILE-LEU-OH**
Xenin 18–25	H-**HIS-PRO-LYS-ARG-PRO-TRP-ILE-LEU-OH**
Xenin 18–25 Gln	H—**HIS-PRO-**GLN-GLN**-PRO-TRP-ILE-LEU-OH**

Common sequences indicated by **bold** typeface

### *In vitro* insulin secretion

BRIN-BD11 cells were used to assess the insulin releasing activity of native xenin, xenin 18–25 and xenin 18–25 Gln [18]. Cells were cultured in RPMI-1640 growth media supplemented with 10% (v/v) foetal bovine serum (FBS) and 1% (v/v) antibiotics (penicillin (100 U/ml), streptomycin (0.1 mg/l)), in 75 cm^2^ sterile tissue culture flasks (Greiner Bio-One, UK) maintained at 37°C and 5% CO_2_ in a LEEC incubator (Laboratory technical engineering, Nottingham, UK). BRIN-BD11 cells were then seeded at a density of 150,000 cells/well in 24-well plates (Nunc, Roskilde, Denmark) and allowed to attach overnight at 37°C. Culture medium was removed and cells were pre-incubated in Krebs–Ringer bicarbonate buffer (KRBB) (115 mmol/l NaCl, 4.7 mmol/l KCl, 1.2 mmol/l MgSO_4_, 1.28 mmol/l CaCl_2_, 1.2 mmol/l KH_2_PO_4_, 25 mmol/l HEPES and 8.4% NaHCO_3_, containing 0.5% (w/v) BSA, pH 7.4) supplemented with 1.1 mmol/l glucose for 40 min at 37°C. Following the pre-incubation, experiments (n = 8 replicates) were performed in presence of glucose (5.6 mM) with a range of concentrations of test peptides (10^−12^ to 10^−6^ mol/l) for 20 min at 37°C. After test incubations, aliquots of assay buffer were collected from each well and stored at -20°C prior to measurement of insulin by radioimmunoassay [19].

### Animals

Acute animal studies were conducted in male NIH Swiss mice (13–15 weeks old, Harlan Ltd, UK) maintained on a standard rodent maintenance diet (10% fat, 30% protein and 60% carbohydrate, Trouw Nutrition, Cheshire, UK). Prior to commencement of longer term studies, all mice were maintained on a high fat diet (45% fat, 35% carbohydrate and 20% protein, Special Diet Services, Essex, UK) for 14 weeks. This diet resulted in progressive body weight gain and hyperglycaemia. All animals were housed individually in an air-conditioned room at 22 ± 2°C with a 12 h light:12 h dark cycle and had free access to food and water. All animal experiments were carried out in accordance with the UK Animals (Scientific Procedures) Act 1986 and approved by the University of Ulster Animal Ethics Review Committee. All necessary steps were taken to ameliorate any potential animal suffering and animals were sacrificed by lethal inhalation of CO_2_ followed by cervical dislocation.

### Acute *in vivo* effects in lean control mice

For food intake studies, fasted (18 h) mice were given intraperitoneal (i.p) injections of either saline vehicle (0.9% w/v NaCl), xenin 18–25 or xenin 18–25 Gln at a dose of 500 nmol/kg bw. This dose was chosen based on observations that supra-physiological doses of peripherally administered xenin are required to impart appetitive suppressive effects [6,7]. Mice were then allowed free access to normal chow for 120 mins and cumulative food intake measured. For glucose homeostasis and insulin secretory studies, blood glucose and plasma insulin concentrations were measured immediately prior to and 15, 30 and 60 min after i.p. administration of glucose alone (18 mmol/kg bw) or in combination with either xenin 18–25 or xenin 18–25 Gln (each at 25 nmol/kg bw) in non-fasted mice. This dose was chosen based on positive metabolic effects of xenin-based drugs using similar dosing regimens and animal models [8,10].

### Sub chronic *in vivo* studies in high fat fed mice

Twice daily (09:30 and 17:30 h) i.p. injections of saline vehicle (0.9% w/v NaCl), xenin 18–25 or xenin 18–25 Gln (both at 25 nmol/kg bw) were administered for 21 days in high fat mice. Energy intake, body weight, non-fasting blood glucose and plasma insulin concentrations were assessed at 3–6 day intervals during the 21 days. At the end of the treatment period, i.p. glucose tolerance (18 mmol/kg bw), biological response to GIP (18 mmol/kg glucose in combination with native GIP (25 nmol/kg); i.p.) and insulin sensitivity (15 U/kg bw; i.p.) tests were performed. All test solutions were administered in a final volume of 5 ml/kg body weight, at 10:00 h without previous 09:30 h peptide injection. Terminal analysis included measurement of total body fat mass by DEXA scanning (Piximus Densitometer, Inside Outside Sales, Madison WI, USA).

### Biochemical analysis

Blood samples were collected from the cut tip on the tail vein of conscious mice into chilled fluoride/heparin glucose micro-centrifuge tubes (Sarstedt, Numbrecht, Germany) at the time points indicated in the Figs Blood glucose was measured directly using a hand-held Ascencia Contour blood glucose meter (Bayer Healthcare, Newbury, Berkshire, UK). Blood samples were centrifuged using a Beckman microcentrifuge (Beckman Instruments, Galway, Ireland) for 1 min at 13,000 x *g* and stored at -20°C. Plasma and pancreatic insulin were assayed by a modified dextran-coated charcoal radioimmunoassay [19].

### Statistical analysis

Statistical analysis was performed using GraphPad PRISM (Version 5). Results are expressed as means ± SEM and data compared using One-way ANOVA or Two-way repeated measures ANOVA, where appropriate, followed by the Student-Newman-Keuls post-hoc test. Groups of data were considered to be significantly different if p<0.05.

## Results

### *In vitro* studies

**Fig 1:**
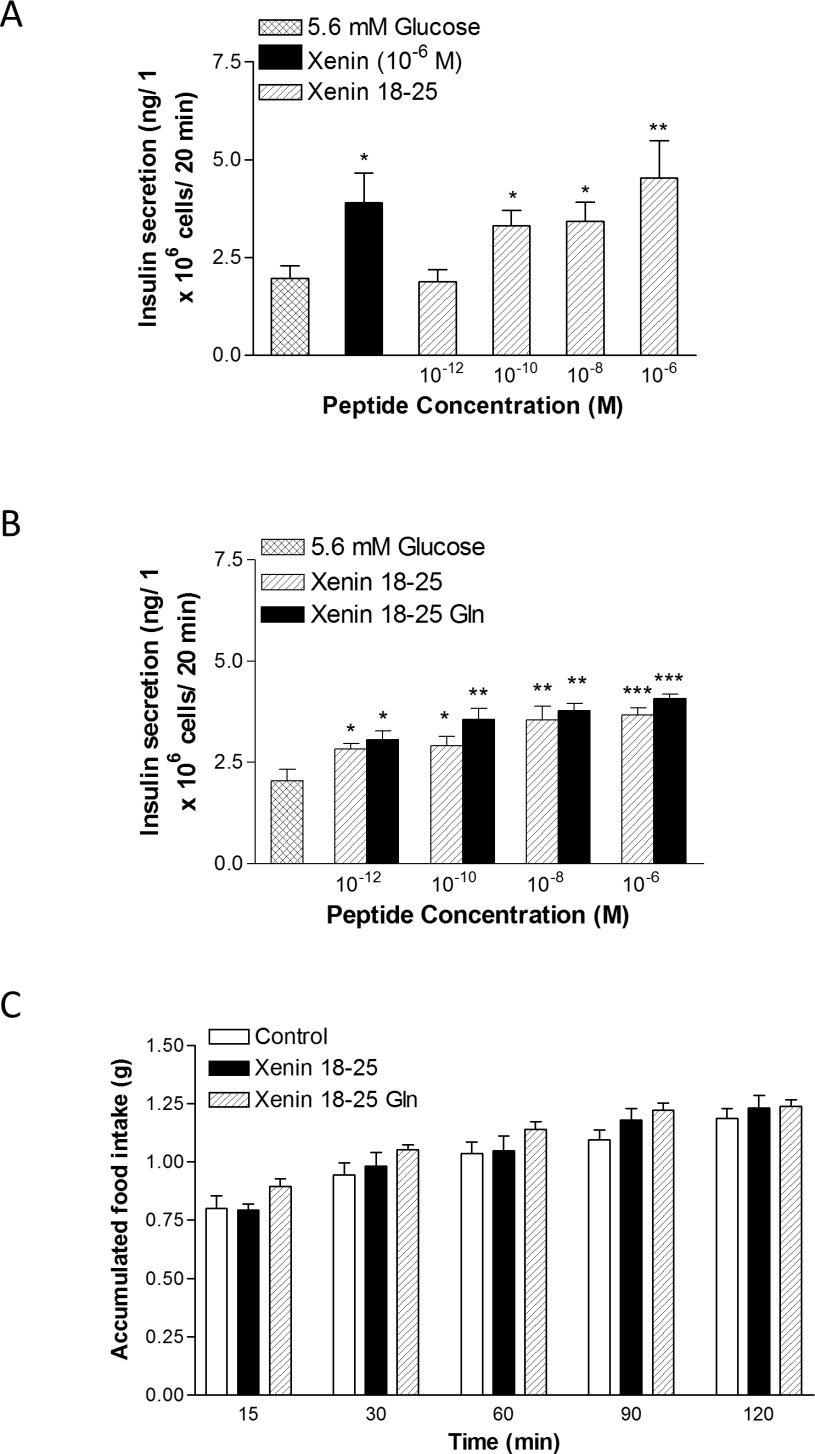
**Effects of native xenin, xenin 18–25 and xenin 18–25 Gln on (A,B) insulin release from BRIN BD11 cells and (C) cumulative food intake in lean control mice.** (A,B) BRIN BD11 cells were incubated (20 min) with a range of concentrations (10^−12^ to 10^−6^ M) of test peptides in the presence of 5.6 mM glucose, and insulin was measured by radioimmunoassay. (C) Cumulative food intake was measured after i.p. injection of saline vehicle (0.9% NaCl), xenin 18–25 or xenin 18–25 Gln (both at 500 nmol/kg bw) in overnight (18 h) fasted mice. Values represent means ± SEM (n = 8). *p<0.05, **p<0.01 and ***p<0.001 compared to 5.6 mM glucose.

Xenin 18–25 significantly (p<0.05 and p<0.01) stimulated insulin secretion from BRIN-BD11 cells compared to 5.6 mM glucose control, with similar efficacy as native xenin (Fig 1A). As shown in Fig 1B, xenin 18–25 Gln had similar, if not slightly enhanced, insulin secretory effectiveness when compared to xenin 18–25.

### Acute *in vivo* studies

**Fig 2:**
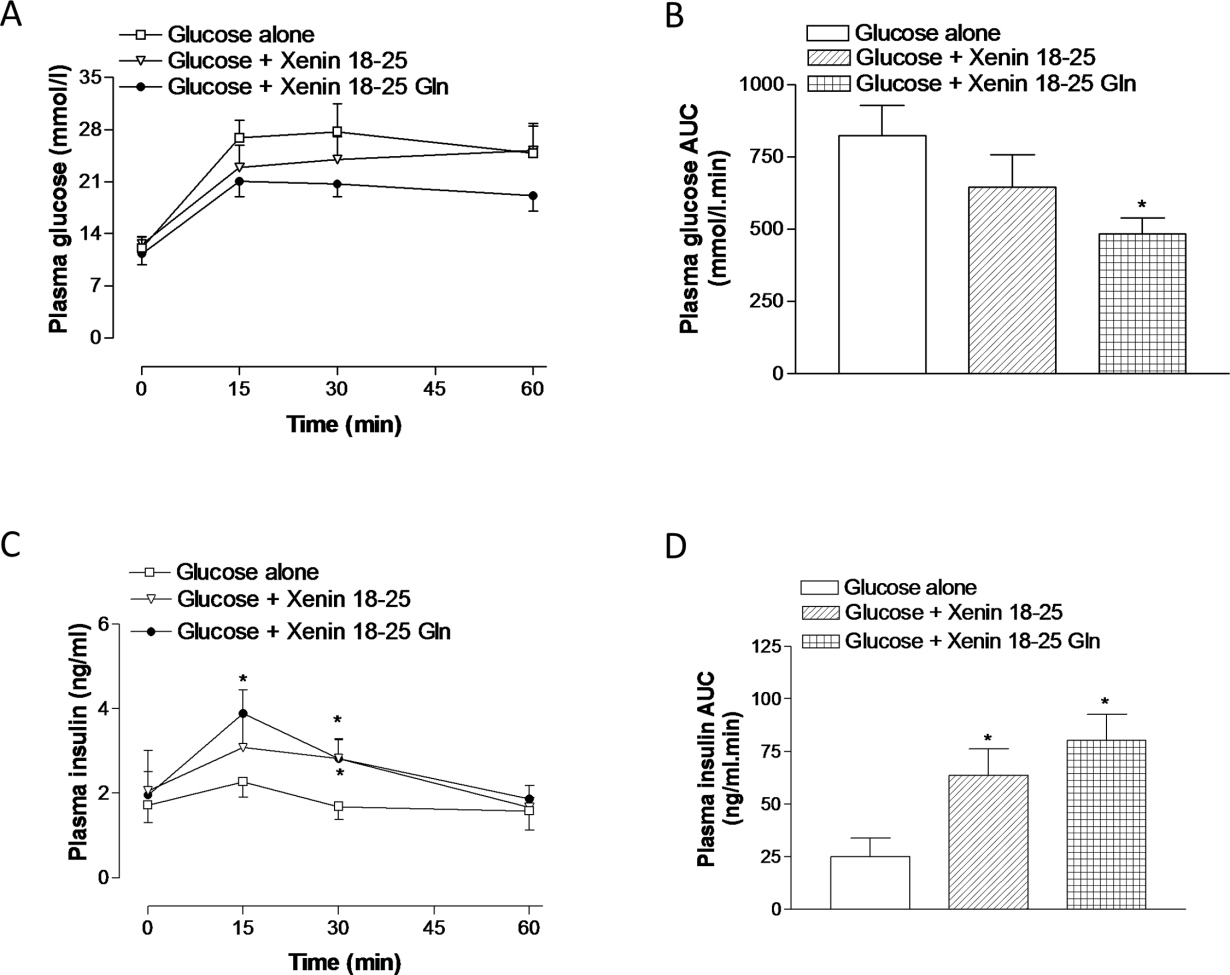
**Glucose lowering (A,B) and insulin releasing (C,D) effects of xenin 18–25 and xenin 18–25 Gln in lean control mice.** (A) Blood glucose and (C) plasma insulin concentrations were measured before and after intraperitoneal injection of glucose alone (18 mmol/kg bw), or in combination with xenin 18–25 or xenin 18–25 Gln (each at 25 nmol/kg bw) in non-fasted mice. (B,D) Blood glucose and plasma insulin AUC values for 0–60 min are also shown. Values represent means ± SEM for 7–8 mice. *p<0.05 compared to glucose alone control.

Xenin 18–25 and xenin 18–25 Gln had no significant satiety effects, even at a supra-pharmacological dose of 500 nmol/kg (Fig 1C). Administration of xenin 18–25 or xenin 18–25 Gln concomitantly with glucose in normal mice resulted in a moderate, although non-significant, lowering of individual blood glucose values (Fig 2A, group: F = 3.829, p<0.05; time: F = 16.53, p<0.001; interaction: F = 0.4794, p>0.05). This culminated in significantly (p<0.05) decreased overall 0–60 min AUC blood glucose concentrations in xenin 18–25 Gln treated mice compared to glucose alone controls (Fig 2B). Corresponding glucose-induced plasma insulin concentrations were elevated (p<0.05) in xenin 18–25 mice at 15 min post-injection, and in xenin 18–25 Gln treated mice at 15 and 30 min post-injection (Fig 2C, group: F = 3.336, p<0.05; time: F = 4.165, p<0.05; interaction: F = 0.5605, p>0.05). In agreement, overall glucose-stimulated plasma insulin levels, as assessed by AUC measures, were significantly (p<0.05) increased in both treatment groups when compared to controls (Fig 2D).

### Effects of twice daily administration of xenin 18–25 and xenin 18–25 Gln on body weight, energy intake, non-fasting blood glucose and plasma insulin in high fat fed mice

**Fig 3:**
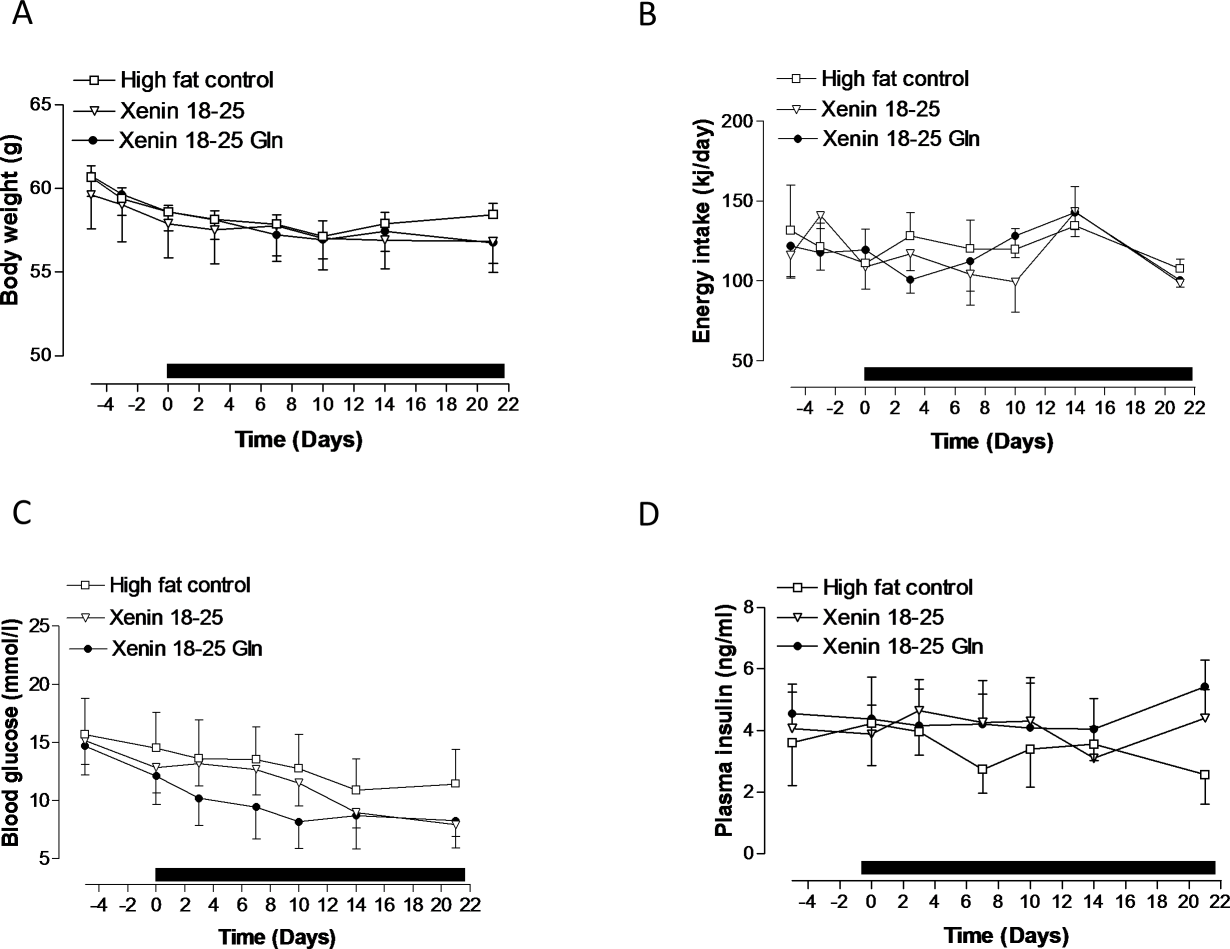
**Effects of twice-daily administration of xenin 18–25 and xenin 18–25 Gln on (A) body weight, (B) energy intake, (C) non fasting blood glucose and (D) non fasting plasma insulin in high fat fed mice.** Parameters were measured for 4 days before and 21 days during (indicated by black horizontal bar) twice daily intraperitoneal injection of saline vehicle (0.9% (w/v) NaCl), xenin 18–25 or xenin 18–25 Gln (each at 25 nmol/kg bw). Values represent means ± SEM for 6–8 mice. *p<0.05 compared to high fat controls.

Twice daily administration of xenin 18–25 and xenin 18–25 Gln for 21 days to high fat fed mice had no significant effect on energy intake or body weight compared to high fat controls (Fig 3A and 3B, group: F = 0.4578, p>0.05; time: F = 1.842, p>0.05; interaction: F = 0.0714, p>0.05). In addition, although non-fasting blood glucose levels were steadily reduced by treatment with both xenin 18–25 and xenin 18–25 Gln, this failed to reach significance over the 21 day period (Fig 3C, group: F = 2.521, p>0.05; time: F = 2.183, p<0.05; interaction: F = 0.1501, p>0.05). In addition, high fat fed mice treated twice daily with xenin 18–25 Gln had noticeable, albeit non-significant, elevations of plasma insulin concentrations compared to saline treated controls on day 21 (Fig 3D).

### Effects of twice daily administration of xenin 18–25 and xenin 18–25 Gln on glucose tolerance and metabolic response to GIP in high fat fed mice

**Fig 4:**
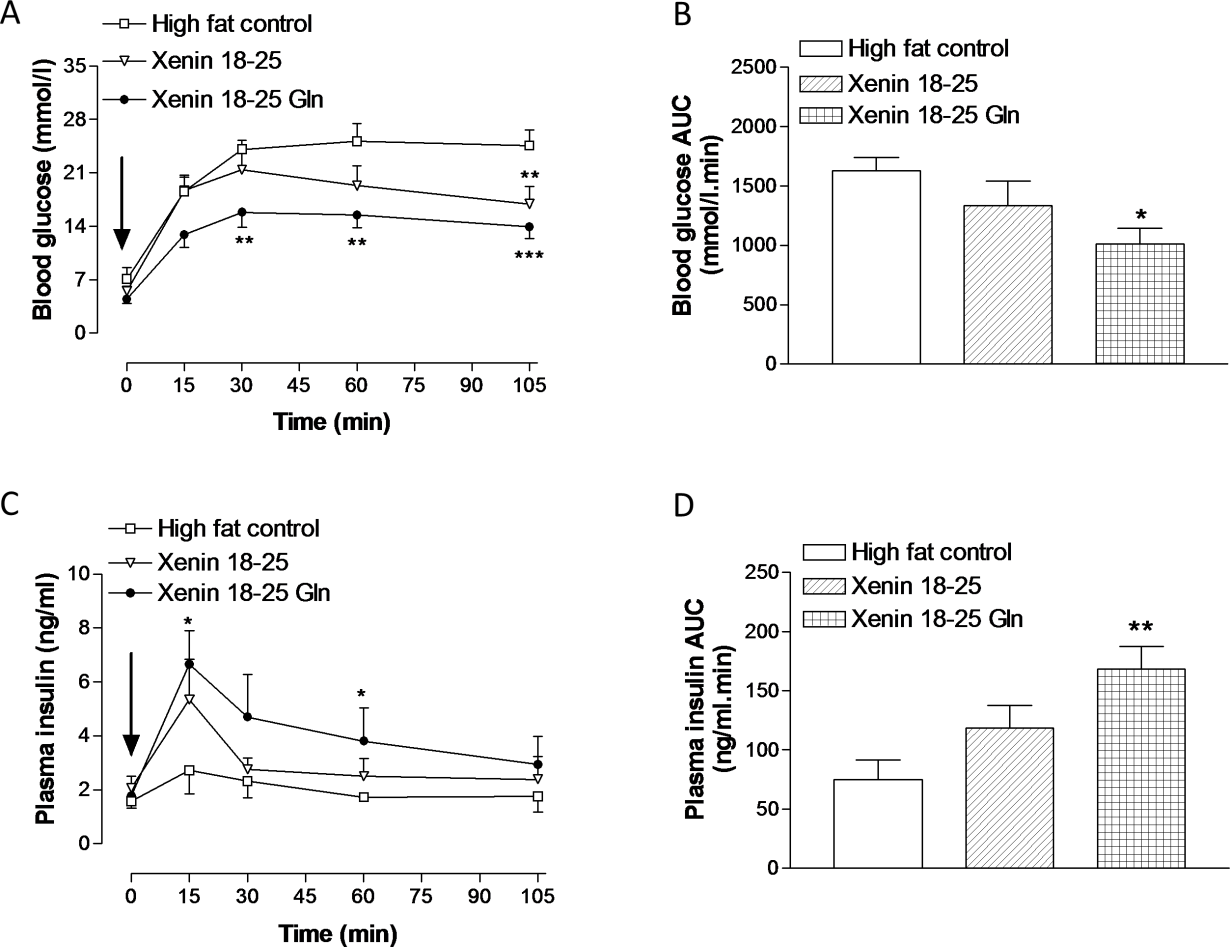
**Effects of twice-daily administration of xenin 18–25 and xenin 18–25 Gln on glucose tolerance (A,B) and glucose-stimulated insulin release (C,D) in high fat fed mice.** Glucose (18 mmol/kg bw) was injected i.p. (t = 0) in overnight fasted mice following 21 days intraperitoneal injection of saline vehicle (0.9% (w/v) NaCl), xenin 18–25 or xenin 18–25 Gln (each at 25 nmol/kg bw). (B,D) Blood glucose and plasma insulin AUC values for 0–105 min are also shown. Values represent means ± SEM for 6–8 mice. *p<0.05, **p<0.01, ***p<0.001 compared to high fat controls.

**Fig 5:**
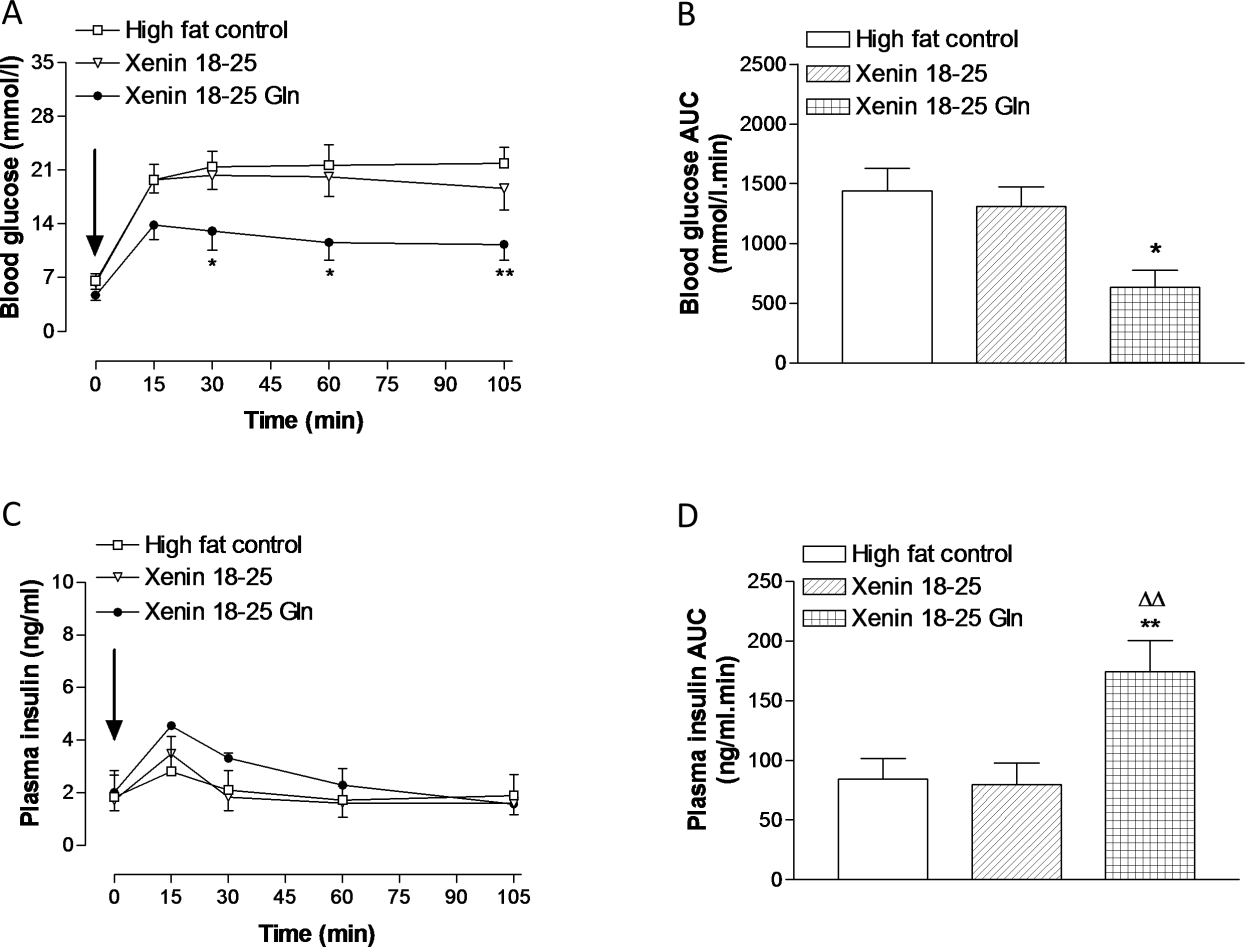
Effects of twice-daily administration of xenin 18–25 and xenin 18–25 Gln on metabolic response to GIP in high fat fed mice. (A,C) Glucose (18 mmol/kg bw) in combination with native GIP (25 nmol/kg bw) was injected i.p. (t = 0) in overnight fasted mice following 21 days intraperitoneal injection of saline vehicle (0.9% (w/v) NaCl), xenin 18–25 or xenin 18–25 Gln (each at 25 nmol/kg bw). (B,D) Blood glucose and plasma insulin AUC values for 0–105 min are also shown. Values represent means ± SEM for 6–8 mice. *p<0.05, **p<0.01 compared to high fat controls. ^Δ^p<0.05 compared to xenin 18–25 group.

Treatment with xenin 18–25, and particularly xenin 18–25 Gln, for 21 days in high fat fed mice significantly (p<0.01) reduced individual blood glucose levels following a glucose load (Fig 4A, group: F = 18.13, p<0.0001; time: F = 30.84, p<0.0001; interaction: F = 1.077, p>0.05). In agreement, blood glucose AUC values were reduced in both xenin 18–25 and xenin 18–25 Gln treated mice, with statistical significance (p<0.05) in the xenin 18–25 Gln treated mice (Fig 4B). Similarly, corresponding glucose-stimulated plasma insulin concentrations were significantly (p<0.05 to p<0.01) elevated, both in terms of individual observation points (Fig 4C, group: F = 3.759, p<0.05; time: F = 3.744, p<0.01; interaction: F = 0.5228, p>0.05) and overall AUC insulin secretory response (Fig 4D), in xenin 18–25 Gln treated mice when compared to saline treated high fat controls (Fig 4C and 4D). As illustrated in Fig 5, xenin 18–25 Gln treatment significantly improved the overall glucose lowering (p<0.05 to p<0.01) actions of native GIP (Fig 5A, group: F = 31.31, p<0.0001; time: F = 10.98, p<0.0001; interaction: F = 1.433, p>0.05), with substantial, but non-significant, elevations of plasma insulin (Fig 5C). As such, the beneficial effects of xenin 18–25 Gln treatment were clearly evident from overall glucose-stimulated plasma insulin AUC values, which were significantly (p<0.01) increased compared to both saline and xenin 18–25 treated high fat fed mice (Fig 5D). In addition, xenin 18–25 Gln treated mice possessed a noticeable and significantly (p<0.05) reduced overall glycaemic excursion compared to high fat controls (Fig 5B). Twice daily treatment with xenin 18–25 had no significant beneficial effects of the glucose-lowering or insulin secretory effects of native GIP (Fig 5A–5D).

### Effects of twice daily administration of xenin 18–25 and xenin 18–25 Gln on insulin sensitivity and percentage body fat mass in high fat fed mice

**Fig 6:**
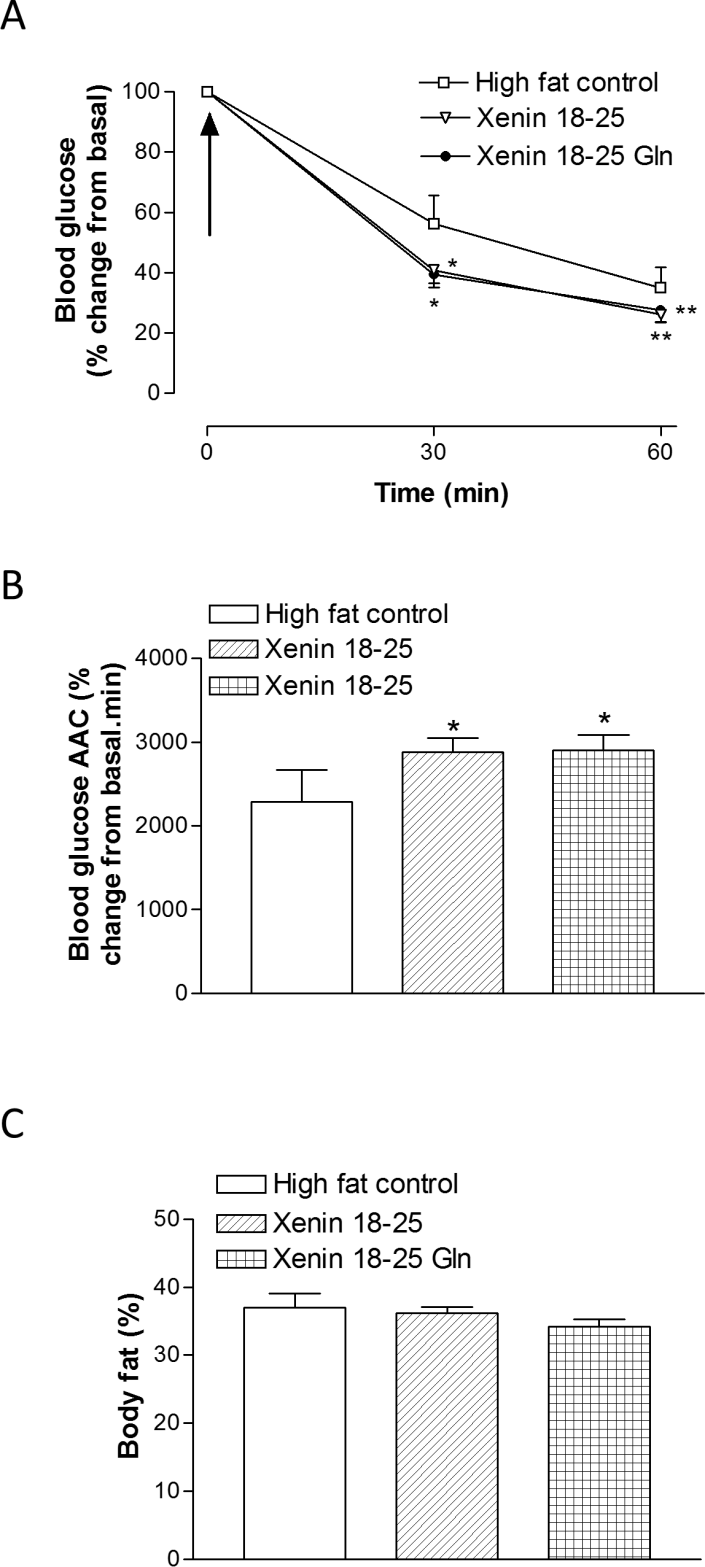
**Effects of twice-daily administration of xenin 18–25 and xenin 18–25 Gln on (A,B) insulin sensitivity and (C) percentage body fat content in high fat fed mice.** (A) Insulin (15 U/kg bw) was injected i.p. (t = 0) in non-fasted mice following 21 days intraperitoneal injection of saline vehicle (0.9% (w/v) NaCl), xenin 18–25 or xenin 18–25 Gln (each at 25 nmol/kg bw). (B) Blood glucose AAC values for 0–60 min are also shown. (C) Total body fat mass was assessed by DEXA scanning on day 21. Values represent means ± SEM for 6–8 mice. *p<0.05, **p<0.01 compared to high fat controls.

Individual blood glucose levels were significantly (p<0.01) reduced at 30 and 60 min post insulin injection in xenin 18–25 and xenin 18–25 Gln treated mice on day 21 compared to high fat controls (Fig 6A, Group: F = 3.32, p<0.05; time: F = 243.6, p<0.0001; interaction: F = 1.138, p>0.05). In agreement, the overall glucose lowering effect of insulin was significantly (p<0.05) improved in both treatment groups (Fig 6B). Percentage body fat mass, as assessed by DEXA scanning, was not significantly different between all groups of high fat mice on day 21 (Fig 6C).

## Discussion

Consistent with previous studies, xenin evoked a prominent stimulation of insulin secretion from clonal pancreatic BRIN-BD11 beta-cells [6,7]. More pertinently, the C-terminal octapeptide fragments, xenin 18–25 and xenin 18–25 Gln, also stimulated *in vitro* insulin secretion, with similar efficacy as the parent peptide. This confirms that both xenin fragment peptides retained full ability to activate xenin related beta-cell signalling pathways that lead to insulin secretion [6,13]. In harmony with this, plasma enzyme degradation analysis of native xenin confirmed that xenin 18–25 was the only degradation fragment of native xenin to possess biological activity [6]. Nonetheless, it should be acknowledged that the definitive mechanism of xenin-induced insulin secretion still requires full elucidation, although the action of phospolipase C has been suggested in this regard [7]. Notably, our observation of pronounced insulin secretory effects of both xenin 18–25 and xenin 18–25 Gln, following conjoint injection with glucose to lean control mice, confirms insulinotropic effectiveness of both compounds.

Based on initial positive *in vitro* and acute *in vivo* data, and in view of the potential therapeutic value of xenin-based drugs for type 2 diabetes [7–10,14], the effects of 21 days treatment with xenin 18–25 and xenin 18–25 Gln were studied in high fat fed mice. In agreement with earlier work using full length enzymatically stable versions of the parent peptide [8,10,14], twice daily injection of high fat mice with xenin 18–25 or xenin 18–25 Gln had no obvious adverse or toxic effects. In fact, food intake and body weight were similar to saline treated control mice. This is in accord with observed lack of effect of both fragment peptides on acute feeding behaviour in the current study, even at supra-pharmacological doses of 500 nmol/kg. In addition, we have previously shown no effect of xenin 18–25 on feeding behaviour in mice, using a similar elevated dosing regimen [6]. However, it must be acknowledged that lack of detrimental effect of the xenin fragment peptides on feeding behaviour in the acute setting does not preclude possible longer-term toxic effects, although similar observations with chronic administration of the peptides is encouraging. Other studies using peripheral administration of native xenin have demonstrated inhibition of food intake in rodents [7,20] and chicks [2]. In this regard, it has recently been revealed that xenin induces appetite suppressive effects through delayed gastric emptying [9] and activation of cells in the nucleus of the solitary tract [21,22]. As such, our findings may indicate lack of passage of xenin 18–25 and xenin 18–25 Gln through the blood brain barrier, however further studies are required to confirm this.

In agreement with prominent insulin secretory actions of xenin based compounds [7,10,13], twice daily treatment with the xenin fragment peptides augmented circulating insulin concentrations by day 21 in high fat fed mice. This beneficial effect was much more prominent with xenin 18–25 Gln, than xenin 18–25. In keeping with substantially improved pancreatic beta-cell dynamics in xenin 18–25 Gln mice, the overall insulin secretory effects, and subsequent glucose-lowering actions, of exogenously delivered glucose, alone and in combination with native GIP, were substantially enhanced in these mice. In type 2 diabetes there is a well characterised global defect of beta-cell insulin secretory capacity that extends to all insulin secretagogoues including glucose [23], and particularly GIP [24]. Indeed, impaired insulin secretory effectiveness of GIP is now recognised as a specific and important pathophysiological characteristic of type 2 diabetes [25]. Thus, it is would be credible to link a major part of the positive therapeutic effects of xenin 18–25 Gln directly to augmentation of GIP action. In accord with this, xenin has previously been shown to significantly potentiate the biological actions of GIP [7,8,10,26]. Notably, the improvement of GIP action by xenin 18–25 Gln in the present study was not related to decreased glucose toxicity, since basal glucose levels were not significantly altered when compared to saline treated high fat control mice. As such, normalisation of blood glucose levels has also been shown to restore GIP insulin secretory function in both rodents and humans with in type 2 diabetes [27,28].

Interestingly, significant insulin-induced reductions of blood glucose levels were observed in both xenin 18–25 and xenin 18–25 Gln treated high fat fed mice, which contrasts with studies using a stable acylated version of the parent peptide [8]. Development of a specific assay to directly measure xenin 18–25 as well as xenin 18–25 Gln in plasma would be useful to determine the pharmacokinetic profile of the fragment peptides. However, it is clear that these xenin peptides induce beneficial metabolic actions that are additional to the observed positive effects on pancreatic beta-cell function. Thus, the observed improvements of glucose tolerance and metabolic response to GIP are likely due to both improved insulin secretion and action. Body fat stores were not altered by either therapy, ruling out the possibility that improvements of insulin sensitivity were simply a consequence of reduced adiposity or altered body composition [29]. Further are required to fully assess the contribution of non-beta cell actions to the overall beneficial effects of xenin 18–25, and particularly xenin 18–25 Gln, such as effects on glucagon secretion or inhibition of gastric emptying [9,13]. In addition, assessment of ambulatory activity or metabolic rate in these animals would also be helpful to uncover the biological consequence of sustained administration of the xenin fragment peptides.

In conclusion, the present study has characterised the bioactivity of two xenin C-terminal fragment peptides, namely xenin 18–25 and xenin 18–25 Gln. The results show that sustained administration of both peptides, and especially xenin 18–25 Gln, to high fat fed mice recapitulated similar beneficial metabolic effects observed with stable forms of the parent peptide [8,14]. This included improved insulin secretory actions, glucose tolerance and metabolic response to exogenous GIP administration. Taken together, our data suggest that the C-terminal octapeptide fragment of xenin represents an important sequence in terms of bioactivity of the molecule, and represents an ideal basis for development of future xenin-based compounds for the potential treatment of type 2 diabetes.
